# RNA-seq reveals altered gene expression levels in proximal tubular cell cultures compared to renal cortex but not during early glucotoxicity

**DOI:** 10.1038/s41598-020-67361-3

**Published:** 2020-06-25

**Authors:** Linnéa M. Nilsson, Miguel Castresana-Aguirre, Lena Scott, Hjalmar Brismar

**Affiliations:** 1 Science for Life Laboratory, Department of Applied Physics, Royal Institute of Technology, Box 1031, 171 21 Solna, Sweden; 2 Science for Life Laboratory, Department of Biochemistry and Biophysics, Stockholm University, Solna, Sweden; 3 Science for Life Laboratory, Department of Women’s and Children’s Health, Karolinska Institutet, Solna, Sweden

**Keywords:** Bioinformatics, RNA sequencing, Diabetes complications

## Abstract

Cell cultures are often used to study physiological processes in health and disease. It is well-known that cells change their gene expression in vitro compared to in vivo, but it is rarely experimentally addressed. High glucose is a known trigger of apoptosis in proximal tubular cells (PTC). Here we used RNA-seq to detect differentially expressed genes in cultures of primary rat PTC, 3 days old, compared to cells retrieved directly from rat outer renal cortex and between PTC exposed to 15 mM glucose and control for 8 h. The expression of 6,174 genes was significantly up- or downregulated in the cultures of PTC compared to the cells in the outer renal cortex. Most altered were mitochondrial and metabolism related genes. Gene expression of proapoptotic proteins were upregulated and gene expression of antiapoptotic proteins were downregulated in PTC. Expression of transporter related genes were generally downregulated. After 8 h, high glucose had not altered the gene expression in PTC. The current study provides evidence that cells alter their gene expression in vitro compared to in vivo and suggests that short-term high glucose exposure can trigger apoptosis in PTC without changing the gene expression levels of apoptotic proteins.

## Introduction

Cell cultures, both primary and immortalized, are often used as models in biological research to investigate physiological processes in health and disease. It is commonly known that cells change their gene expression in vitro compared to in vivo. Yet the extent of the differentiation is not fully understood. We therefore wanted to compare gene expression in primary cell cultures, 3 days old, with cells retrieved directly from the kidney. Our group has previously used primary rat proximal tubule cells (PTC) to investigate the intrinsic apoptotic pathway in diseases such as proteinuric kidney disease^1^ and hemolytic uremic syndrome^2^. In this study, we chose to examine changes in gene expression between primary PTC and tissue from outer renal cortex, which volume mostly consists of proximal tubule^3^. The main aim of this study was to discern in which groups of genes that the gene expression level is most altered in primary PTC cultures compared to cells in the outer renal cortex. In addition, we wanted to investigate if gene expressions related to our previous studies had significantly changed within 3 days.

Hyperglycemia is one of the most common symptoms in diabetic kidney disease, where PTC have been identified as one of the targets of glucotoxicity^4^. Our group have previously used PTC to study the apoptotic response to short-term high glucose exposure^5^. In addition to study changes in gene expression in PTC compared to outer renal cortex, we therefore decided to study whether gene expression levels would change in PTC exposed to 15 mM glucose for 8 h compared to control. If that was the case, we wanted to identify which groups of genes had changed their expression the most.

## Methods

### Cell culture and tissue preparation

Twenty-day-old male Sprague Dawley rats were used for preparation of proximal tubule slices and PTC cultures. All animals were housed under controlled conditions of light and dark (12:12 h) and given a standard diet containing 20% protein by weight and tap water were available ad libitum. All experiments were performed according to Karolinska Institutet regulations concerning care and use of laboratory animals and were approved by the Stockholm North ethical evaluation board for animal research.

Proximal tubule slices were collected from the outer 150 µm of the renal cortex, where 90% of the tubular volume is proximal tubules^3^. Primary cultures of rat PTC were prepared as previously described^1^ using the outer 150 µm renal cortex as starting material. Cells were seeded in 60-mm wells and cultured in 37 °C at an approximate humidity of 95–98% with 5% CO_2_ for 3 days before experiments. Culture medium was changed every 24 h. Cells were exposed to 15 mM glucose (HG) or 5.6 mM glucose (control) for 8 h. Kidney cortex samples were prepared in replicates from three animals. PTC samples were prepared from three separate cultures and pairwise exposed to HG or control.

### RNA-seq

Cells and tissue samples were collected and mRNA extracted and purified with RNeasy mini kit (cat. no. 74134, Qiagen AB, Sollentuna, Sweden) following manufacturer’s instructions. The quality of the starting RNA was validated with an Agilent Bioanalyzer before cDNA libraries were created. The cDNA libraries were created by National Genomics Infrastructure at Science for Life Laboratory (Solna, Sweden) using Illumina TruSeq Stranded mRNA with poly-A selection. Each sample was used to generate two separate cDNA libraries. Quality controls of the libraries were performed by National Genomics Infrastructure at Science for Life Laboratory (Solna, Sweden) using MultiQC.

### Bioinformatics analyses

Differential expression analysis was performed with the R package edge R^6^ and heatmaps were created using the R package pheatmap. The cDNA libraries were aligned to a reference genome, which was created using the *Rattus norvegicus* genome from National Center for Biotechnology Information webpage^7^. The annotations for each gene was retrieved from National Center for Biotechnology Information webpage^7^ and matched to each gene start and stop codon position. The gene symbols were added from the R package org.Rn.eg.db^8^.

Gene symbols occasionally appeared in the list of genes more than once. Only the gene transcript with the highest number of counts for each gene was saved. The list of genes was filtered with the edgeR function *filterByExpr.* We required the genes to have at least 10 counts in one sample and at least a total of 20 counts across all samples to be included in the analysis. These requirements were fulfilled by 7,615 genes. We performed trimmed mean of M-value normalization to remove possible composition bias between samples.

**Figure 1:**
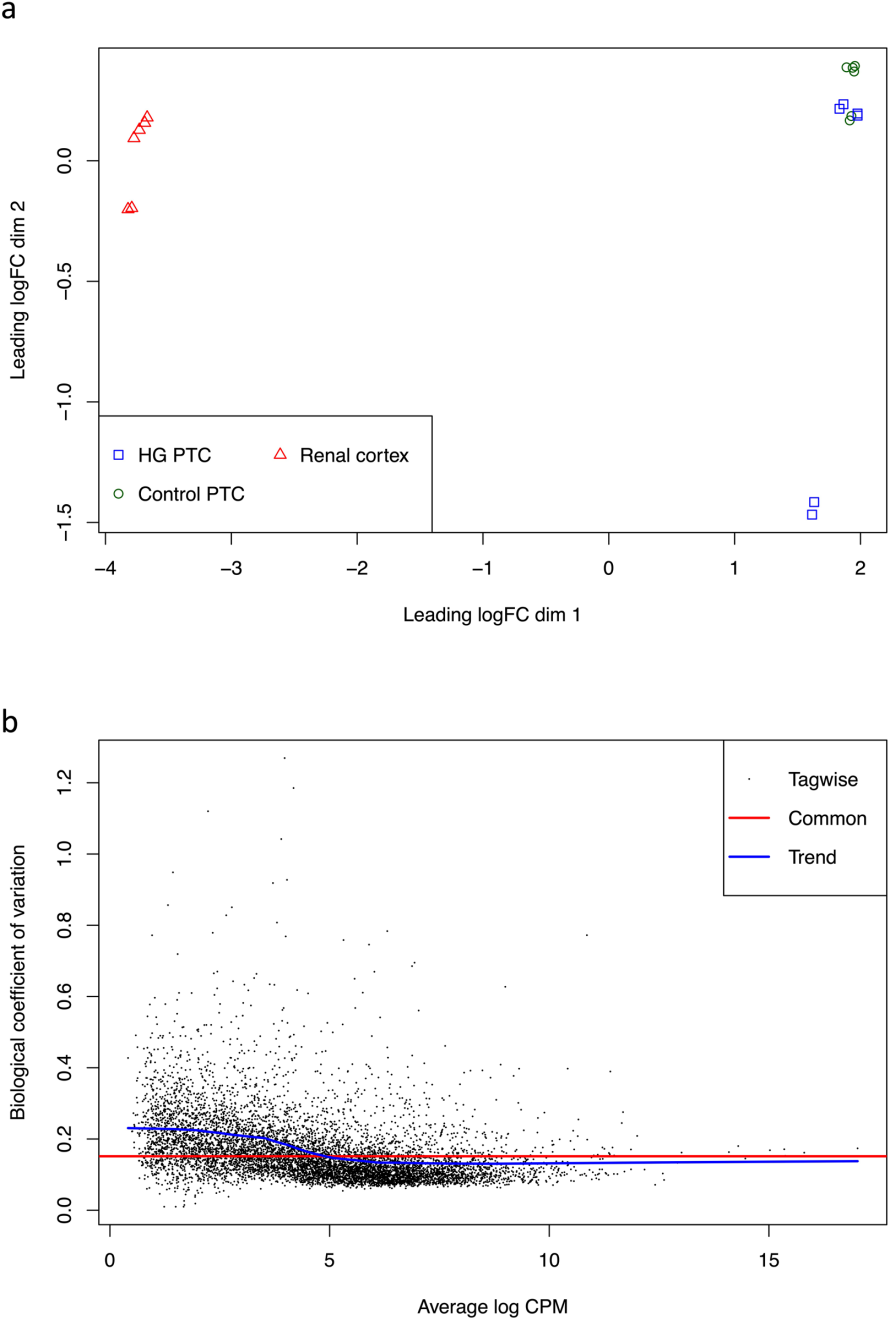
(**a**) Multi-dimensional scaling plot showing differences in gene expression profile between the samples. Differences in days in culture, i.e. between PTC and renal cortex, are visualized horizontally and differences in treatment, i.e. between control and HG, are visualized vertically. In red: renal cortex samples. In green: control PTC samples. In blue: HG PTC samples. (**b**) Biological coefficient of variation of all the samples. In black: the tagwise dispersions for each gene. In red: the common dispersion. In blue: the trend dispersion.

Differences between the expression profiles of the samples were visualized with a multi-dimensional scaling plot (Fig. 1a). The plot shows a large difference in gene expression profile between renal cortex and PTC and a small difference between PTC incubated in control and HG medium for 8 h. The fold-change (FC) between renal cortex and PTC was ~ 2^6^ = 64 and the FC between control and HG exposed PTC was within 2^0.5^ ≈ 1.4, except for one HG sample where the FC was ~ 2^1.5^ ≈ 2.8 from control samples. The common negative binomial dispersion among the samples was estimated to approximately 0.023 and the biological coefficient of variation is shown in Fig. 1b.

### Bax and Bcl-xl abundance assessment

Abundance of Bax and Bcl-xL was assessed as previously described^5^. Briefly, 3 days old PTC were incubated with control or HG for 8 h. Cells were fixed with 4% paraformaldehyde (pH 7.4) for 10 min, permeabilized with 0.3% Triton X-100 for 10 min and blocked with 5% BSA in 0.1% Triton X-100 for 1 h. Primary antibodies mouse monoclonal anti-Bax (6A7) (5 μg/ml) (Abcam, Cambridge, UK) and rabbit monoclonal anti-Bcl-x_L_ (54H6) (1:200) (Cell Signaling Technology, Danvers, MA, USA) were applied over night at 4 °C. Cells were washed and secondary antibodies Alexa Fluor 546 goat anti-mouse IgG (Life Technologies, Carlsbad, CA, USA) and Alexa Fluor 546 goat anti-rabbit IgG (Life Technologies, Carlsbad, CA, USA) were applied for 1 h at room temperature. Secondary antibody controls were subjected to the same treatment, but primary antibodies were omitted. Cells were imaged with a Zeiss LSM 510 confocal microscope equipped with × 63/1.4 NA oil objective. The microscope setting was kept fixed for all measurements. The Bax and Bcl-xL abundances were analyzed in Matlab (The MathWorks, Natick, MA, USA). The total abundance of Bax and Bcl-xL was calculated as the percentage of Bax or Bcl-xL (pixels) normalized to cell size (pixels). On each coverslip, at least three cells were analyzed. The control group was set to 100%.

### Statistics

Statistical significance of the differential expression analysis was determined with a one-way ANOVA for each gene using the glmQLFTest function in edge R. The significance of differentially expressed genes was determined by false discovery rate (FDR). A FDR < 0.05 was considered significant.

## Results

### Mitochondrial and metabolism GO terms were most altered in PTC compared to renal cortex

We first screened for differentially expressed genes in PTC cultures compared to outer renal cortex slices. The expression of 3,042 genes was significantly downregulated in PTC compared to renal cortex and it was significantly upregulated for 3,132 genes. To identify the groups of genes that were overrepresented in PTC compared to renal cortex we performed a gene ontology (GO) enrichment analysis. Mitochondrial and metabolism related GO terms, including mitochondrion (GO-CC:0005739), oxidation–reduction process (GO-BP:0055114) and drug metabolic process (GO-BP:0017144) were among the uppermost overrepresented GO terms in PTC compared to renal cortex (Table 1). The expression of the genes in the top GO terms were generally downregulated. Next, we performed pathway enrichment analysis on KEGG pathway database to identify which pathways were altered in PTC compared to renal cortex. The most significantly involved pathways for our studied conditions were metabolic (adjusted p < 1.70e−42) and oxidative phosphorylation (adjusted p < 1.65e−31) pathways (SI Table S1), which confirms the result of the GO enrichment analysis.

**Table 1:** The top 10 GO terms overrepresented in PTC cultures compared to renal cortex slices.

No	GOID	Term	N	Up	Down	P.up	P.down
1	GO-CC:0044429	Mitochondrial part	456	51	361	1	1.24e−65
2	GO-CC:0005739	Mitochondrion	773	140	536	1	3.46e−64
3	GO-CC:0098798	Mitochondrial protein complex	185	3	173	1	3.79e−51
4	GO-CC:0005740	Mitochondrial envelope	320	41	246	1	6.28e−39
5	GO-CC:0031966	Mitochondrial membrane	293	37	227	1	1.35e−36
6	GO-CC:0005743	Mitochondrial inner membrane	194	11	165	1	2.37e−35
7	GO-CC:0044455	Mitochondrial membrane part	150	6	135	1	2.70e−34
8	GO-BP:0044281	Small molecule metabolic process	831	220	503	1	5.43e−33
9	GO-BP:0055114	Oxidation–reduction process	487	124	323	1	5.19e−30
10	GO-CC:0019866	Organelle inner membrane	217	24	173	1	6.53e−30

*No* order in list of overrepresented GO terms, *N* total number of genes associated with the GO term, *Up* number of upregulated genes within the GO term, *Down* number of downregulated genes within the GO term, *P.up* p value of upregulated genes after adjustment with the Bonferroni correction method, *P.down* p value of downregulated genes after adjustment with the Bonferroni correction method, *CC* cellular component, *BP* biological process.

It is commonly known that an altered environment can initiate changes of cytoskeletal proteins in cells such as podocytes, causing dedifferentiation^9^. In this study, we found that this was also the case for primary cultures of PTC. Cytoskeleton GO terms such as cytoskeleton organization (GO-BP:0007010) and cytoskeleton (GO-CC:0005856) were significantly overrepresented in PTC compared to renal cortex (SI Table S2).

Our group has previously reported an altered balance between pro- and antiapoptotic proteins in PTC exposed to toxic levels of albumin, Shiga toxin and HG^1,2,5^. Glucotoxic-triggered apoptosis may be a consequence of a changed glucose metabolism and transport under hyperglycemic condition^5^. In the current study we therefore investigated if an altered environment would induce a change in genes related to cell death, glucose metabolism and transporter activity. Apoptosis related GO terms, including regulation of apoptotic process (GO-BP:0042981), regulation of programmed cell death (GO-BP:0043067) and apoptotic process (GO-BP:0006915) were a bit down in the list of overrepresented GO terms (at 585–673) (Table 2). General transporter related GO terms such as transmembrane transporter activity (GO-MF:0022857) were among the top 100 overrepresented GO terms in PTC compared to renal cortex (at 70–88) (Table 3). The glucose related GO terms; glucose transmembrane transporter activity (GO-MF:0005355) and positive regulation of glucose metabolic process (GO-BP:0010907), were in the end of the list of overrepresented GO terms (at 2,428–2,644) (Table 3). The expression of most genes in the transporter related GO terms were downregulated in PTC compared to renal cortex.

**Table 2:** The top 5 apoptosis related GO terms overrepresented in PTC cultures compared to renal cortex slices.

No	GOID	Term	N	Up	Down	P.up	P.down
584	GO-BP:0042981	Regulation of apoptotic process	670	323	245	0.631	1
632	GO-BP:0043067	Regulation of programmed cell death	676	324	248	1	1
638	GO-BP:0006915	Apoptotic process	784	371	291	1	1
643	GO-BP:0043065	Positive regulation of apoptotic process	303	156	102	1	1
672	GO-BP:0010941	Regulation of cell death	743	352	276	1	1

*No* order in list of overrepresented GO terms, *N* total number of genes associated with the GO term, *Up* number of upregulated genes within the GO term, *Down* number of downregulated genes within the GO term, *P.up* p value of upregulated genes after adjustment with the Bonferroni correction method, *P.down* p value of downregulated genes after adjustment with the Bonferroni correction method, *BP* biological process.

**Table 3:** Glucose metabolism and transport related GO terms overrepresented in PTC cultures compared to renal cortex slices.

No	GOID	Term	N	Up	Down	P.up	P.down
69	GO-MF:0022857	Transmembrane transporter activity	343	96	206	1	1.18e−10
82	GO-BP:0055085	Transmembrane transport	549	175	302	1	9.69e−10
87	GO-MF:0005215	Transporter activity	417	125	239	1	1.35e−09
2427	GO-MF:0005355	Glucose transmembrane transporter activity	13	3	9	1	1
2643	GO-BP:0010907	Positive regulation of glucose metabolic process	17	11	4	1	1

*No* order in list of overrepresented GO terms, *N* total number of genes associated with the GO term, *Up* number of upregulated genes within the GO term, *Down* number of downregulated genes within the GO term, *P.up* p value of upregulated genes after adjustment with the Bonferroni correction method, *P.down* p value of downregulated genes after adjustment with the Bonferroni correction method, *MF* molecular function, *BP* biological process.

**Figure 2:**
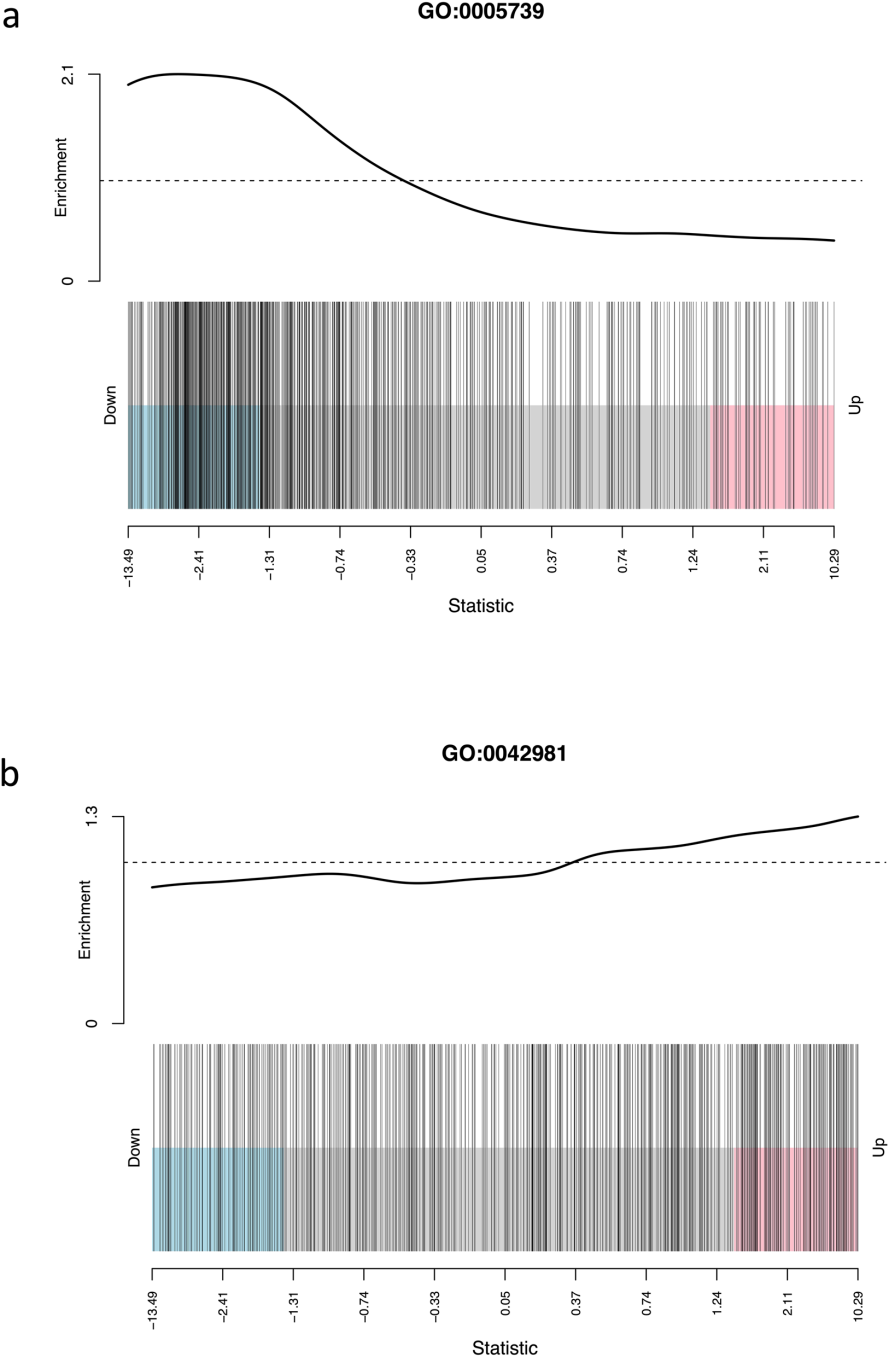
(**a**) Representative barcode plot showing up- and downregulated genes in the GO term mitochondrion (GO-CC:0005739). Each line represents one gene. Genes within the GO term is predominantly downregulated in PTC compared to renal cortex. (**b**) Representative barcode plot showing up- and downregulated genes in the GO term regulation of apoptotic process (GO-BP:0042981). Each line represents one gene. Genes within the GO term is predominantly upregulated in PTC compared to renal cortex.

Barcode plots were created for each GO term to observe the range of up- and downregulated genes. The mitochondrial and metabolism related GO terms had more genes with downregulated expression in PTC compared to renal cortex (Fig. 2a), whereas apoptosis related GO terms had slightly more genes with upregulated expression (Fig. 2b).

### Differential expression analysis reveals altered expression level of mitochondrial, metabolism, cytoskeleton, apoptosis and transporter genes in PTC

We next identified the top differentially expressed genes in the overrepresented GO terms. In the mitochondrial and metabolism related GO terms from Table 2, the expression of genes such as *Pck1*, *Dao*, *Amacr*, *Ndufv2* and *Prodh2* were among the most significantly downregulated in PTC compared to renal cortex and the gene expression of *Loxl2* was among the most significantly upregulated (Table 4). Cytoskeletal genes were also differentially expressed. Most significantly upregulated were *Cnn2*, *Eppk1*, *Myh9l1* and *Myh9* and most significantly downregulated were *Msrb1* and *Pink1* (SI Table S3). In the apoptosis related GO terms from Table 3, the most significantly upregulated genes were *Gpx1*, *Hpgd*, *Prdx5* and *Aqp2* and most significantly downregulated genes were *Plk3*, *Casp12*, *Notch2* and *Map3k20* (Table 5). Interestingly, *Bcl2*, *Tmbim6* and *Casp2* were among the top 100 differentially expressed apoptosis related genes. *Bcl2* and *Tmbim6* were downregulated in PTC compared to renal cortex, whereas the gene expression of *Casp2* was upregulated. Many transporters were differentially expressed in PTC compared to renal cortex (Tables 6, 7), including the gene expression of *Aqp1/2/3/6*, *Naglt1*, *Slc5a1* and *Slc2a2,* which were downregulated, and *Slc2a1*, which was upregulated. The gene expression of *Slc5a2*, *Atp1a1* and *Atp1b1* were not among the top differentially expressed transporter genes, but were still significantly downregulated in PTC compared to renal cortex.

**Table 4:** The top 15 differentially expressed genes in the GO terms in the Table 2 in PTC compared to renal cortex.

Symbol	Gene name	Entrezid	logFC	p value	FDR
*Pck1*	Phosphoenolpyruvate carboxykinase 1	362282	− 11.9	5.90e−28	2.55e−24
*Loxl2*	Lysyl oxidase-like 2	290350	6.72	1.15e−26	2.91e−23
*Dao*	d-Amino-acid oxidase	114027	− 9.10	8.90e−26	1.70e−22
*Amacr*	Alpha-methylacyl-CoA racemase	25284	− 5.33	6.14e−25	6.68e−22
*Ndufv2*	NADH:ubiquinone oxidoreductase core subunit V2	81728	− 3.40	8.09e−25	7.17e−22
*Prodh2*	Proline dehydrogenase 2	361538	− 7.57	2.10e−24	1.33e−21
*Ndufa6*	NADH:ubiquinone oxidoreductase subunit A6	315167	− 3.55	3.15e−24	1.85e−21
*Acat1*	Acetyl-CoA acetyltransferase 1	25014	− 3.85	3.86e−24	2.10e−21
*Ech1*	Enoyl-CoA hydratase 1	64526	− 3.39	4.26e−24	2.16e−21
*Pah*	Phenylalanine hydroxylase	24616	− 9.14	6.16e−24	2.61e−21
*Acaa2*	Acetyl-CoA acyltransferase 2	170465	− 4.34	7.22e−24	2.88e−21
*Cyp2e1*	Cytochrome P450, family 2, subfamily e, polypeptide 1	25086	− 13.5	7.94e−24	2.88e−21
*Glyatl2*	Glycine-*N*-acyltransferase-like 2	171179	− 9.81	1.14e−23	3.78e−21
*Atp5po*	ATP synthase peripheral stalk subunit OSCP	192241	− 3.62	1.28e−23	4.05e−21
*Miox*	Myo-inositol oxygenase	252899	− 12.3	1.46e−23	4.44e−21

The symbol, gene name, entrezid, log fold-change (FC), p value and false discovery rate (FDR) for each gene as indicated. Positive logFC indicate a higher gene expression in PTC compared to renal cortex and vice versa.

**Table 5:** The top 15 differentially expressed genes in the apoptosis related GO terms from Table 3 in PTC compared to renal cortex.

Symbol	Gene name	Entrezid	logFC	p value	FDR
*Gpx1*	Glutathione peroxidase 1	24404	− 3.68	3.97e−23	8.94e−21
*Hpgd*	15-Hydroxyprostaglandin dehydrogenase	79242	− 6.61	4.88e−22	6.28e−20
*Prdx5*	Peroxiredoxin 5	113898	− 2.49	2.36e−21	2.06e−19
*Scp2*	Sterol carrier protein 2	25541	− 3.08	1.43e−20	8.79e−19
*Plk3*	Polo-like kinase 3	58936	5.50	2.66e−20	1.50e−18
*Casp12*	Caspase 12	156117	5.13	2.73e−20	1.52e−18
*Notch2*	Notch 2	29492	2.66	6.97e−20	3.11e−18
*Aqp2*	Aquaporin 2	25386	− 10.5	7.19e−20	3.18e−18
*Cyr61*	Cysteine-rich, angiogenic inducer, 61	83476	4.42	7.53e−20	3.30e−18
*Klk1*	Kallikrein 1	24523	− 11.3	2.26e−19	8.08e−18
*Myc*	MYC proto-oncogene, bHLH transcription factor	24577	4.27	4.32e−19	1.45e−17
*Dusp1*	Dual specificity phosphatase 1	114856	2.96	6.19e−19	1.95e−17
*Map3k20*	Mitogen-activated protein kinase kinase kinase 20	311743	2.11	9.03e−19	2.70e−17
*Atp5f1a*	ATP synthase F1 subunit alpha	65262	− 2.53	1.02e−18	3.01e−17
*Folh1*	Folate hydrolase 1	85309	− 7.28	1.91e−18	5.19e−17

The symbol, gene name, entrezid, log fold-change (FC), p value and false discovery rate (FDR) for each gene as indicated. Positive logFC indicate a higher gene expression in PTC compared to renal cortex and vice versa.

**Table 6:** The top 15 differentially expressed genes in the GO terms; transmembrane transporter activity (GO-MF:0022857), transmembrane transporter (GO-BP:0055085) and transporter activity (GO-MF:0005215) from Table 4 in PTC compared to renal cortex.

Symbol	Gene name	Entrezid	logFC	p value	FDR
*Slc22a8*	Solute carrier family 22 member 8	83500	− 10.8	2.07e−23	5.85e−21
*Naglt1*	Na + dependent glucose transporter 1	337920	− 7.53	3.24e−23	8.22e−21
*Spns2*	Sphingolipid transporter 2	100270678	5.61	1.42e−22	2.40e−20
*Aqp1*	Aquaporin 1	25240	− 7.11	4.95e−22	6.28e−20
*Aqp6*	Aquaporin 6	29170	− 8.73	9.26e−22	9.84e−20
*Slc13a3*	Solute carrier family 13 member 3	64846	− 5.98	1.80e−21	1.63e−19
*Slc25a5*	Solute carrier family 25 member 5	25176	− 2.3	2.64e−20	1.50e−18
*Aqp2*	Aquaporin 2	25386	− 10.5	7.19e−20	3.18e−18
*Slc22a25*	Solute carrier family 22, member 25	192273	− 4.79	2.03e−19	7.42e−18
*Slc6a19*	Solute carrier family 6 member 19	664630	− 7.70	8.36e−19	2.52e−17
*Abcd3*	ATP binding cassette subfamily D member 3	25270	− 1.86	2.96e−18	7.42e−17
*Slc22a5*	Solute carrier family 22 member 5	29726	− 2.63	3.68e−18	8.91e−17
*Aqp3*	Aquaporin 3	65133	− 8.45	4.06e−18	9.75e−17
*Sec61a1*	Sec61 translocon alpha 1 subunit	80843	1.62	2.30e−17	4.33e−16
*Slc6a20*	Solute carrier family 6 member 20	113918	− 5.50	2.45e−17	4.55e−16

The symbol, gene name, entrezid, log fold-change (FC), p value and false discovery rate (FDR) for each gene as indicated. Positive logFC indicate a higher gene expression in PTC compared to renal cortex and vice versa.

**Table 7:** The top 10 differentially expressed genes in the GO term glucose transmembrane transporter activity (GO-MF:0005355) from Table 4 in PTC compared to renal cortex.

Symbol	Gene name	Entrezid	logFC	p value	FDR
*Naglt1*	Na + dependent glucose transporter 1	337920	− 7.53	3.24e−23	8.22e−21
*RGD1310495*	Similar to KIAA1919 protein	309809	− 6.51	3.19e−21	2.53e−19
*Slc5a1*	Solute carrier family 5 member 1	25552	− 6.78	1.38e−19	5.30e−18
*RGD1304770*	Similar to Na + dependent glucose transporter 1	309810	− 4.94	9.52e−17	1.50e−15
*Slc2a1*	Solute carrier family 2 member 1	24778	2.00	2.59e−15	2.82e−14
*RGD1561777*	Similar to Na + dependent glucose transporter 1	499463	− 5.09	2.67e−15	2.89e−14
*Slc45a1*	Solute carrier family 45, member 1	246258	8.50	9.54e−11	3.86e−10
*Slc2a5*	Solute carrier family 2 member 5	65197	− 2.82	4.53e−07	9.98e−07
*Slc2a2*	Solute carrier family 2 member 2	25351	− 1.24	1.55e−06	3.17e−06
*LOC100909595*	Solute carrier family 2, facilitated glucose transporter member 3-like	100909595	2.07	1.53e−05	2.78e−05

The symbol, gene name, entrezid, log fold-change (FC), p value and false discovery rate (FDR) for each gene as indicated. Positive logFC indicate a higher gene expression in PTC compared to renal cortex and vice versa.

### High glucose does not alter gene expression levels in PTC during an early state

We identified differentially expressed genes in PTC exposed to HG for 8 h compared to control. Only one genes expression was significantly upregulated in the HG exposed PTC compared to control (FDR < 0.02). No genes expression was significantly downregulated. The gene with significantly upregulated expression was identified as *Ubn2*, which codes for the nuclear protein ubinuclein 2. The function of ubinuclein 2 is still relatively unknown and it remains to be concluded if the function of this protein is relevant for glucotoxicity.

## Discussion

Cell models are often used to study signaling pathways in health and disease in a controlled and isolated environment. It is commonly known, but rarely discussed, that cells may change their gene expression in vitro compared to in vivo. Here we addressed this question using RNA-seq to identify differentially expressed genes between renal outer cortex and 3 days old PTC cultures.

The gene expression level of most genes, 6,174 of 7,615, was significantly altered in the PTC cultures compared to the renal outer cortex slices. We identified the largest changes in gene expression levels in mitochondrial and metabolism related GO terms, which could be indicating that cells in culture have a changed energy expenditure and metabolism compared to cells in the renal cortex. It is known that cells gradually lose their phenotype in vitro. In the current study, the primary cells still expressed the same genes as the renal cortex slices even though the expression level for a large number of genes was significantly up- or downregulated. These data indicate that the differentiation process of cells starts directly after cells are isolated. Primary cells are generally believed to keep their gene expression compared to immortalized cells. The results from the current study therefore opens up questions about to which extent the gene expression of immortalized cells differ from primary cells and from their original tissue. These questions still remain to be determined.

It has previously been shown that the expression of many proteins change when podocytes are cultured in vitro^9^. In particular, it has been demonstrated that the culture conditions, such as the elastic modulus of the substrate, have a strong influence on the expression of proteins related to the actin cytoskeleton, including stress fibers and focal adhesion proteins^10^. In the current study we found that the expressions of cytoskeleton genes were significantly altered in cultures of PTC. This may be an indication that an altered environment initiates a change in PTC morphology, which could be a consequence of PTC losing their polarization in vitro compared to in vivo.

Apoptosis associated GO terms were overrepresented in PTC compared to renal cortex. Differentially expressed genes in the overrepresented apoptosis related GO terms included genes that codes for proteins involved in fibrosis, apoptosis and inflammation. The gene expression of the antiapoptotic proteins Bcl2 (*Bcl2*) and Bax inhibitor 1 (*Tmbim6*) were significantly downregulated in PTC and the gene expression of the executor protein caspase 2 (*Casp2*) was significantly upregulated, suggesting that PTC may have an increased susceptibility to apoptosis in vitro compared to proximal tubules in vivo. The gene expression for the TGFβ superfamily members bone morphogenetic proteins 1, 3 and 5 (*Bmp1/3/5*) were both up- and downregulated in PTC compared to tubule and the gene expression of caspase 12 (*Casp12*) was significantly upregulated. These data suggest a change in fibrosis and inflammation processes, which could be an indication that a healing process has started due to the dissociation of cells in culture compared to in vivo, where cells are less spread out.

The gene expression of many transporters was significantly up- or downregulated in PTC compared to renal cortex, including the gene expressions for the sodium-dependent glucose transporters NaGLT (*Naglt*), SGLT1 (*Slc5a1*) and SGLT2 (*Slc5a2*), which were downregulated, and the gene expression for the glucose transporter GLUT1 (*Slc2a1*), which was upregulated. The gene expression for GLUT2 (*Slc2a2*) was downregulated in PTC compared to renal cortex. We also found that several genes belonging to the solute carrier membrane group (SLC) of transport proteins relevant for transport of other substrates, including amino acids, fatty acids and ions, were also significantly changed (Table 8). Altogether these results indicate that cells in culture change their transport of solutes. In particular is the glucose uptake affected in PTC with a reduced sodium-dependent glucose uptake compared to the cells that express sodium-glucose cotransporters in vivo, in order to accommodate for a lower rate of glucose metabolism.

**Table 8:** The top 15 differentially expressed genes in the Slc family in PTC compared to renal cortex.

Symbol	Gene name	Substrate	logFC	p value	FDR
*Slc34a1*	Sodium/phosphate cotransporter 2A	Sodium/phosphate	−11.1	5.10e−24	2.43e−21
*Slc22a8*	Organic anion transporter 3	Organic anions	−10.8	2.07e−23	5.85e−21
*Slc27a2*	Very long-chain-fatty-acid-CoA ligase	Free long-chain fatty acids	−8.65	9.90e−22	1.02e−19
*Slc13a3*	Sodium/dicarboxylate cotransporter 3	Sodium/Krebs cycle intermediates	−5.98	1.80e−21	1.63e−19
*Slc22a1*	Organic cation transporter 1	Organic cations	−5.91	3.47e−21	2.70e−19
*Slc25a5*	ADP/ATP translocase 2	ADP/ATP	−2.37	2.64e−20	1.50e−18
*Slc44a3*	Choline transporter-like protein 3	Choline	−3.33	4.20e−20	2.06e−18
*Slc38a3*	Sodium-coupled neutral amino acid transporter 3	Sodium/glutamine/protons	−5.61	5.04e−20	2.34e−18
*Slc5a1*	Sodium/glucose cotransporter 1	Sodium/glucose	−6.78	1.38e−19	5.30e−18
*Slc22a25*	Organic anion transporter UST6	Unknown	−4.79	2.03e−19	7.42e−18
*Slc6a19*	Sodium-dependent neutral amino acid transporter B(0)AT1	Sodium/neutral amino acids	−7.70	8.36e−19	2.52e−17
*Slc22a5*	Organic cation/carnitine transporter 2	Sodium/carnitine	−2.63	3.68e−18	8.91e−17
*Slc16a9*	Monocarboxylate transporter 9	Unknown	−5.02	3.91e−18	9.44e−17
*Slc6a20*	Sodium/chloride-dependent transporter XTRP3	Small hydrophilic substrates	−5.50	2.45e−17	4.55e−16
*Slc13a1*	Sodium/sulfate cotransporter	Sodium/sulfate	−4.59	2.90e−17	5.25e−16

The symbol, gene name, substrate(s) of the transporter, log fold-change (FC), p value and false discovery rate (FDR) for each gene as indicated. Positive logFC indicate a higher gene expression in PTC compared to renal cortex and vice versa.

In addition to the downregulation of sodium-dependent glucose transporters, the gene expression of aquaporins (*Aqp1/2/3/6*), Na^+^/K^+^-ATPase subunit α1 (*Atp1a1*) and β1 (*Atp1b1*) were also significantly downregulated. SGLT 1 and 2, aquaporins and Na^+^/K^+^-ATPase are transporters that in vivo facilitates vectorial transport across PTC. In vitro, PTC are no longer polarized, which may contribute to decreased gene expression of transporters that facilitates vectorial transport.

**Figure 3:**
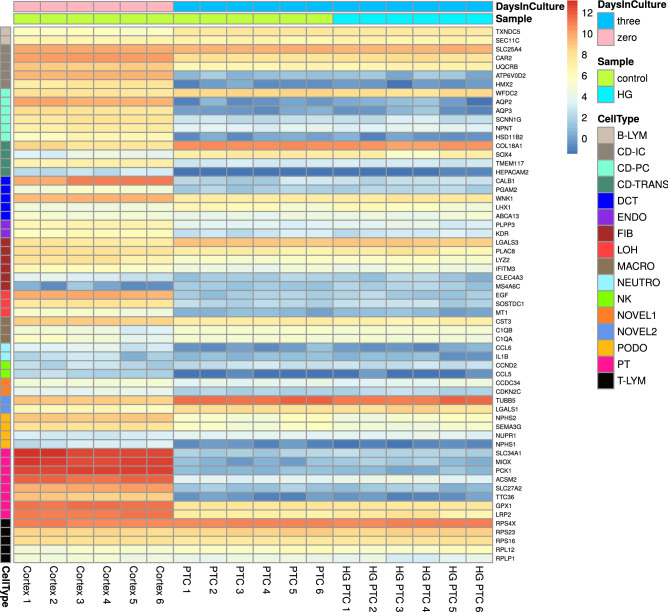
Heatmap for cell-type specific markers^10^. We compared 3 days old cell cultures of primary PTC with cells retrieved directly from the outer renal cortex, and between PTC exposed to 15 mM of glucose and control for 8 h. Genes were clustered by cell-type. Gene expression goes from blue (low expression) to red (high expression). Samples are ordered by days in culture (DaysInCulture), and by control and high glucose (HG).

RNA-seq has previously been used to study gene expressions of the whole kidney^10,11^. To focus the current study on the proximal tubule we instead used thin slices of outer renal cortex. A large number of cell-type specific genes^11^ that are expressed in other kidney cells than PTC were therefore not expressed in our samples. A drawback of the current study is that only 90% of the renal outer cortex slices consist of proximal tubular volume^3^, whereas 99% of PTC are SGLT2-positive when stained with antibodies^1^. The fraction of PTC is thereby higher in PTC samples compared to the renal tissue samples and may have affected the readout of the GO enrichment analysis and differential expression analysis between cells and tissue, especially for PTC specific genes. Differences in kidney cell composition, PTC-specific genes and PTC segment specific genes among the samples is shown in Fig. 3, SI Figs. S1, S2, S3 and S4, respectively, using cell-type and segment specific genes (SI Tables S4–S4)^11,12^. To fully conclude differentially expressed genes in PTC cultures a tissue sample containing only proximal tubule would be required.

**Figure 4:**
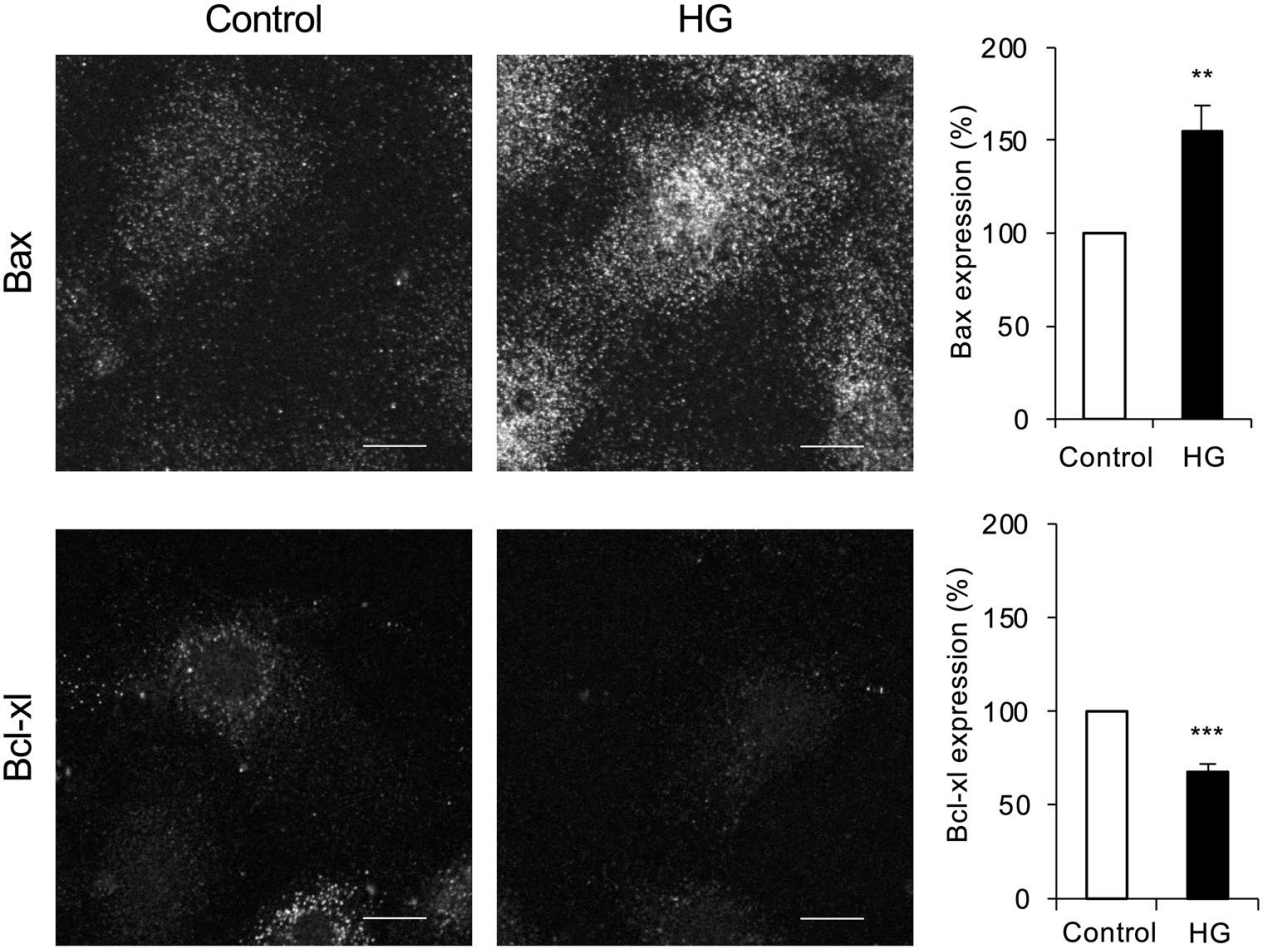
Data revised from our publication Nilsson et al.^5^. Left: representative images of immunostaining for Bax and Bcl-xl in PTC incubated with control or HG containing medium for 8 h. Scale bars are 10 µm. Each image visualizes one cell. Right: quantification of Bax and Bcl-xl protein expression in PTC incubated with control or HG containing medium for 8 h. Data is expressed as mean ± SEM. n = 15 coverslips from 5 individual cell preparations. **p < 0.01, ***p < 0.001.

Other studies have shown that hyperglycemia exerts a change in gene expressions of PTC after 24–48 h of exposure to 25–30 mM glucose^4,13^. Apoptosis related genes were significantly altered after exposure to 25 mM glucose for 48 h. The current study did not find a significant change in gene expression. This is likely due to a shorter exposure to HG. However, the present study only shows a snapshot of what happens after 8 h of glucose exposure. It is therefore not possible to conclude what happens before or after 8 h. To determine how HG effects the gene expression levels in PTC, a time or does response curve might be necessary. The protein expression of the antiapoptotic protein Bcl-xl was however significantly downregulated after 8 h of exposure to HG, while the protein expression of the proapoptotic protein Bax was significantly upregulated (Fig. 4). These data suggest that HG triggers an acute regulation of apoptotic protein levels within 8 h, without regulation of gene expression, which has been reported to be altered after 48 h^4^.

One potential drawback of the current study is that one of the HG samples differed somewhat in FC. The reason for this variation is not known. It may be within the expected biological variation, since primary cells could respond differently to HG exposure. To full conclude how short-term exposure to high glucose affects the gene expression levels it might be necessary to include more than three replicates to better estimate the effects of the biological variance.

In the current study we conclude that genes are differentially expressed in cultured cells compared tissue, which highlights the importance to verify that cells still express the genes of interest when setting up experiments. The results from this study show that PTC still express the same genes as in tubules, but that the gene expression level is altered. Short-term exposure of HG did not significantly alter gene expression levels, which may be a later response to glucotoxicity.

## Supplementary information

Supplementary information 1

Supplementary information 2

Supplementary information 3

Supplementary information 4

## Data Availability

The datasets generated during and/or analyzed during the current study are available in the Datadryad repository, https://doi.org/10.5061/dryad.v9s4mw6rb.

## References

[1] Burlaka I (2016). Prevention of apoptosis averts glomerular tubular disconnection and podocyte loss in proteinuric kidney disease. Kidney Int..

[2] Burlaka I (2013). Ouabain protects against shiga toxin-triggered apoptosis by reversing the imbalance between Bax and Bcl-xL. J. Am. Soc. Nephrol..

[3] Aperia A, Larsson L, Zetterström R (1981). Hormonal induction of Na-K-ATPase in developing proximal tubular cells. Am. J. Physiol..

[4] Ortiz A, Ziyadeh FN, Neilson EG (1997). Expression of apoptosis-regulatory genes in renal proximal tubular epithelial cells exposed to high glucose ambient glucose and in diabetic kidney. Kidney Int..

[5] Nilsson LM (2019). Prompt apoptotic response to high glucose in SGLT expressing renal cells. Am. J. Physiol. Renal Physiol..

[6] Robinson MD, McCarthy DJ, Smyth GK (2010). edgeR: A Bioconductor package for differential expression analysis of digital gene expression data. Bioinformatics.

[7] National Center for Biotechnology Information. *Rattus norvegicus (Norway rat)*. https://www.ncbi.nlm.nih.gov/genome?term=rattus%20norvegicus (2019).

[8] Carlson, M. *org.Rn.eg.db: Genome wide annotation for Rat. R Package Version 3.8.2*. 10.18129/B9.bioc.org.Rn.eg.db (2019).

[9] Rinschen MM (2016). Quantitative deep mapping of the cultured podocyte proteome uncovers shifts in proteostatic mechanisms during differentiation. Am. J. Physiol. Cell Physiol..

[10] Rinschen MM (2018). A multi-layered quantitative in vivo expression atlas of the podocyte unravels kidney disease candidate genes. Cell Rep..

[11] Park J (2018). Single-cell transcriptomics of mouse kidney reveals potential cellular targets of kidney disease. Science.

[12] Clark JZ (2019). Representation and relative abundance of cell-type selective markers in whole-kidney RNA-Seq data. Kidney Int..

[13] Feliers D, Kasinath BS (2010). Mechanism of VEGF expression by high glucose in proximal tubule epithelial cells. Mol. Cell Endocrinol..

