# Supplementary material for: RNA-seq reveals altered gene expression levels in proximal tubular cell cultures compared to renal cortex but not during early glucotoxicity
Source: Sci Rep. 2020 Jun 25;10:10390. doi: 10.1038/s41598-020-67361-3 (PMC7316724; doi:10.1038/s41598-020-67361-3)
Supplement: Supplementary file 1 — Supplementary information 1 [file 41598_2020_67361_MOESM1_ESM.pdf]

SUPPLEMENTARY INFORMATION

RNA-seq reveals altered gene expression levels in proximal tubular cell cultures compared to renal cortex but not during early glucotoxicity

Linnéa M. Nilsson[1], Miguel Castresana-Aguirre[2], Lena Scott[3], and Hjalmar Brismar[1,3*]

[1]Science for Life Laboratory, Department of Applied Physics, Royal Institute of Technology, Solna, Sweden

[2]Science for Life Laboratory, Department of Biochemistry and Biophysics, Stockholm University, Solna, Sweden

[3]Science for Life Laboratory, Department of Women's and Children's Health, Karolinska Institutet, Solna, Sweden

Pathway	N	Up	Down	P.up	P.down
Metabolic pathways	713	169	462	1	1.70e-42
Oxidative phosphorylation	101	2	97	1	1.65e-31
Parkinson disease	100	7	90	1	2.57e-23
Huntington disease	118	21	95	1	4.08e-17
Thermogenesis	140	26	105	1	6.62e-15
Alzheimer disease	104	14	82	1	1.38e-13
Non-alcoholic fatty liver disease (NAFLD)	91	19	68	1	4.34e-09
Peroxisome	58	3	48	1	7.63e-09
Carbon metabolism	68	12	52	1	2.77e-07
Ribosome	118	13	78	1	2.19e-06
Valine, leucine and isoleucine degradation	31	2	28	1	2.24e-06
Fatty acid degradation	27	1	25	1	4.54e-06
Retrograde endocannabinoid signaling	63	8	47	1	7.23e-06
Glycine, serine and threonine metabolism	26	1	24	1	1.06e-05
Citrate cycle (TCA cycle)	20	1	19	1	1.06e-04
PI3K-Akt signaling pathway	141	87	34	1.78e-04	1
MAPK signaling pathway	114	73	25	1.88e-04	1
Pathways in cancer	228	128	60	8.26e-04	1
MicroRNAs in cancer	78	52	16	1.39e-03	1
Hepatocellular carcinoma	82	54	17	1.60e-03	1
Arrhythmogenic right ventricular cardiomyopathy (ARVC)	31	25	4	2.60e-03	1
Hepatitis B	72	48	15	3.25e-03	1
Regulation of actin cytoskeleton	91	58	25	3.35e-03	1
Propanoate metabolism	19	2	17	1	3.60e-03
Cardiac muscle contraction	34	7	26	1	5.23e-03
Axon guidance	71	47	12	5.34e-03	1
TNF signaling pathway	53	37	14	7.09e-03	1
Yersinia infection	55	38	10	7.99e-03	1
Hippo signaling pathway	68	45	13	8.17e-03	1
Chronic myeloid leukemia	43	31	6	1.22e-02	1
IL-17 signaling pathway	36	27	7	1.24e-02	1
Rheumatoid arthritis	44	12	31	1	1.29e-02
Small cell lung cancer	47	33	9	1.65e-02	1
Glyoxylate and dicarboxylate metabolism	17	2	15	1	1.82e-02
Hypertrophic cardiomyopathy (HCM)	32	24	4	3.39e-02	1
Collecting duct acid secretion	13	1	12	1	4.42e-02
Systemic lupus erythematosus	44	6	30	1	4.50e-02
Focal adhesion	83	51	23	4.59e-02	1

Supplemental Table 1. KEGG pathways overrepresented in PTC cultures compared to renal cortex slices. No – order in list of overrepresented GO terms, N – total number of genes associated with the GO term, Up – number of upregulated genes within the GO term, Down – number of downregulated genes within the GO term, P.up – pvalue of upregulated genes after adjustment with the Bonferroni correction method, P.down – pvalue of downregulated genes after adjustment with the Bonferroni correction method.

No	GOID	Term	N	Up	Down	P.up	P.down
**96**	GO-BP:0007010	Cytoskeleton organization	527	296	142	4.17e-09	1
**156**	GO-CC:0005856	Cytoskeleton	759	394	226	1.80e-06	1
**167**	GO-CC:0044430	Cytoskeletal part	600	319	173	4.00e-06	1
**215**	GO-CC:0099513	Polymeric cytoskeletal fiber	234	139	67	8.32e-05	1
**300**	GO-MF:0005200	Structural constituent of cytoskeleton	27	24	1	3.42e-03	1
**308**	GO-BP:0030036	Actin cytoskeleton organization	270	151	80	4.35e-03	1
**319**	GO-MF:0008092	Cytoskeletal protein binding	360	193	111	6.79e-03	1
**366**	GO-CC:0015629	Actin cytoskeleton	182	106	50	1.87e-02	1

Supplemental Table 2. The top 10 GO terms overrepresented in PTC cultures compared to renal cortex slices. No – order in list of overrepresented GO terms, N – total number of genes associated with the GO term, Up – number of upregulated genes within the GO term, Down – number of downregulated genes within the GO term, P.up – pvalue of upregulated genes after adjustment with the Bonferroni correction method, P.down – pvalue of downregulated genes after adjustment with the Bonferroni correction method, BP – biological process, CC – cellular component, MF – molecular function.

Symbol	Gene name	Entrezid	logFC	p-value	FDR
Cnn2	Calponin 2	690976	4.52	2.39e-22	3.64e-20
Eppk1	Epiplakin 1	680860	6.23	3.05e-22	4.39e-20
Myh9l1	Myosin, heavy chain 9, non-muscle-like 1	25745	3.86	9.13e-22	9.84e-20
Myh9	Myosin, heavy chain 9, non-muscle	100911597	3.55	2.46e-21	2.13e-19
Msrb1	Methionine sulfoxide reductase B1	685059	-3.51	3.10e-21	2.48e-19
Map1b	Microtubule-associated protein 1B	29456	5.13	1.93e-20	1.16e-18
Arhgef5	Rho guanine nucleotide exchange factor 5	140898	2.23	3.94e-20	1.96e-18
Capn2	Calpain 2	29154	1.95	8.97e-20	3.77e-18
Tubb4a	Tubulin, beta 4A class IVa	29213	4.28	1.25e-19	4.90e-18
Rhou	Ras homolog family member U	678766	3.34	1.84e-19	6.78e-18
Tpm4	Tropomyosin 4	24852	2.97	2.64e-19	9.29e-18
Amotl1	Angiomotin-like 1	315430	3.63	2.97e-19	1.03e-17
Pink1	PTEN induced putative kinase 1	298575	-4.94	5.14e-19	1.66e-17
Cdh1	Cadherin 1	83502	3.20	7.83e-19	2.40e-17
Map3k20	Mitogen-activated protein kinase kinase kinase 20	311743	2.11	9.03e-19	2.70e-17

Supplemental Table 3. The top 15 differentially expressed genes in the GO terms of Supplemental Table 2 in PTC compared to renal cortex. The symbol, gene name, entrezid, log fold-change (FC), p-value and false discovery rate (FDR) for each gene as indicated. Positive logFC indicate a higher gene expression in PTC compared to renal cortex and vice versa.

Gene	Cell-type	In heatmap	Gene	Cell-type	In heatmap
Plat	Endo	NO	Fxyd4	CD-PC	NO
Emcn	Endo	NO	Apela	CD-PC	NO
Plpp1	Endo	NO	Aqp3	CD-PC	YES
Ehd3	Endo	NO	Npnt	CD-PC	YES
Nrp1	Endo	NO	Scnn1b	CD-PC	NO
Ly6c1	Endo	NO	Wfdc2	CD-PC	YES
Egfl7	Endo	NO	Kcne1	CD-PC	NO
Meis2	Endo	NO	Scnn1g	CD-PC	YES
Kdr	Endo	YES	Atp6v1g3	CD-IC	NO
Plpp3	Endo	YES	Atp6v0d2	CD-IC	YES
Nphs2	Podo	YES	Hmx2	CD-IC	YES
Podxl	Podo	NO	Car2	CD-IC	YES
H2-Q7	Podo	NO	Oxgr1	CD-IC	NO
Nupr1	Podo	YES	Mme	CD-IC	NO
Cdkn1c	Podo	NO	Tmem61	CD-IC	NO
Nphs1	Podo	YES	Slc25a4	CD-IC	YES
Sparc	Podo	NO	Fam13a	CD-IC	NO
Sema3g	Podo	YES	Uqcrb	CD-IC	YES
Clic3	Podo	NO	Slc26a4	CD-Trans	NO
Rasl11a	Podo	NO	Car12	CD-Trans	NO
Miox	PT	YES	Rhbg	CD-Trans	NO
Slc34a1	PT	YES	Hepacam2	CD-Trans	YES
Akr1c21	PT	NO	Insrr	CD-Trans	NO
Ttc36	PT	YES	Clcnkb	CD-Trans	NO
Slc27a2	PT	YES	Col18a1	CD-Trans	YES
Acsm2	PT	YES	Slc43a2	CD-Trans	NO
Ass1	PT	NO	Tmem117	CD-Trans	YES
Gpx1	PT	YES	Sox4	CD-Trans	YES
Pck1	PT	YES	Lockd	Novel1	NO
Lrp2	PT	YES	Mki67	Novel1	NO
Slc12a1	LOH	NO	Cdca3	Novel1	NO
Umod	LOH	NO	Ccdc34	Novel1	YES
Egf	LOH	YES	Spc25	Novel1	NO
Wfdc15b	LOH	NO	Cox6b2	Novel1	NO
Mt2	LOH	NO	Pbk	Novel1	NO
Ppp1r1a	LOH	NO	Hjurp	Novel1	NO
Mt1	LOH	YES	Cdkn2c	Novel1	YES
Sostdc1	LOH	YES	Serpinb8	Novel1	NO
Slc5a3	LOH	NO	Lyz2	Fib	YES
Ly6a	LOH	NO	Plac8	Fib	YES
Slc12a3	DCT	NO	Ifitm3	Fib	YES
Calb1	DCT	YES	Ifi27l2a	Fib	NO
Wnk1	DCT	YES	Lst1	Fib	NO
Pvalb	DCT	NO	Gngt2	Fib	NO
Pgam2	DCT	YES	Ms4a6c	Fib	YES
Wnk4	DCT	NO	S100a4	Fib	NO
Sgms2	DCT	NO	Lgals3	Fib	YES
Slc16a7	DCT	NO	Clec4a3	Fib	YES
Lhx1	DCT	YES	Cd74	Macro	NO
Abca13	DCT	YES	H2-Aa	Macro	NO
Aqp2	CD-PC	YES	H2-Ab1	Macro	NO
Hsd11b2	CD-PC	YES	H2-Eb1	Macro	NO

Gene	Cell-type	In heatmap	Gene	Cell-type	In heatmap
C1qa	Macro	YES	Gimap3	T-lym	NO
C1qb	Macro	YES	Rps16	T-lym	YES
C1qc	Macro	NO	Cxcr6	T-lym	NO
Apoe	Macro	NO	Rps4x	T-lym	YES
Cst3	Macro	YES	Il7r	T-lym	NO
H2-DMa	Macro	NO	Rps23	T-lym	YES
S100a8	Neutro	NO	Gm10260	T-lym	NO
S100a9	Neutro	NO	Rplp1	T-lym	YES
Retnlg	Neutro	NO	Ccl5	NK	YES
Tyrobp	Neutro	NO	Gzma	NK	NO
Il1b	Neutro	YES	Nkg7	NK	NO
Wfdc21	Neutro	NO	Gzmb	NK	NO
Ngp	Neutro	NO	Klrd1	NK	NO
Ccl6	Neutro	YES	Klrk1	NK	NO
S100a6	Neutro	NO	Ccnd2	NK	YES
S100a11	Neutro	NO	Klre1	NK	NO
Jchain	B-lym	NO	Irf8	NK	NO
Slpi	B-lym	NO	Klra4	NK	NO
Mzb1	B-lym	NO	Hmgb2	Novel2	NO
Cd79a	B-lym	NO	Hist1h2ap	Novel2	NO
Cd79b	B-lym	NO	Crip1	Novel2	NO
Ly6d	B-lym	NO	Lgals1	Novel2	YES
Ebf1	B-lym	NO	Ms4a4b	Novel2	NO
Mef2c	B-lym	NO	Coro1a	Novel2	NO
Sec11c	B-lym	YES	Tubb5	Novel2	YES
Txndc5	B-lym	YES	2810417H13Rik	Novel2	NO
Ltb	T-lym	NO	Stmn1	Novel2	NO
Rpl12	T-lym	YES	Arhgdib	Novel2	NO

Supplemental Table 4. List of cell-type specific markers (1) that were included in the analysis to generate the heatmap in Figure 3. A total of 10 genes from each cell-type were included in the analysis, however not all genes were expressed in the samples. For each gene it is indicated which cell-type that express the gene and if the gene is expressed or not in the samples.

Gene	In heatmap	Gene	In heatmap
Miox	YES	Ccdc107	NO
Slc34a1	YES	Ces1f	NO
Akr1c21	NO	Cat	YES
Ttc36	YES	Ugt2b38	NO
Slc27a2	YES	Inmt	YES
Acsm2	YES	4833439L19Rik	NO
Ass1	NO	Slc7a13	YES
Gpx1	YES	Nudt19	NO
Pck1	YES	Fmo2	NO
Lrp2	YES	Ak4	NO
Cela1	YES	Xylb	NO
Guca2b	YES	Slc22a28	NO
Fut9	NO	Pipox	NO
Hrsp12	NO	Neu1	YES
Keg1	NO	Akr7a5	NO
Khk	NO	Macrod2	NO
Calml4	YES	Slc5a8	YES
Dnase1	NO	Slc22a1	YES
Sord	NO	Trim7	NO
Slc4a4	YES	Gpd1	NO
Cml1	NO	Aldh6a1	YES
Glyat	NO	Ghr	NO
Pdzk1	YES	Upb1	YES
Fbp1	YES	Aspdh	YES
Cda	NO	Rab11fip3	NO
Acy3	NO	Slc17a3	YES
Errfi1	NO	Car14	NO
Kcnj15	NO	Slc37a4	NO
Cyp2j5	NO	Gm11128	NO
Snhg11	NO	Slc22a18	NO
Slc22a6	NO	Tcn2	NO
Akr1a1	YES	Gcdh	YES
Slc22a12	NO	D630029K05Rik	NO
4931406C07Rik	NO	Slc7a7	NO
Dnajc12	YES	Gstz1	YES
Cndp2	NO	Tmem150a	NO
Slc47a1	NO	Asl	NO
G6pc	YES	Ldhd	YES
Nox4	NO	Prodh2	YES
Slc17a1	YES	Slc6a18	NO
Slc13a3	YES	0610011F06Rik	NO
Mep1a	YES	Fah	YES
Slc6a19	YES	Prodh	NO
Bdh2	NO	Hspe1	YES
Tnfaip8	NO	Cyp2d26	NO
Scp2	YES	Prss8	YES
Ugt3a2	NO	Ces1d	YES
Tmem174	YES	Gm10804	NO
Slc22a30	NO	Ephx2	YES
Pecr	YES	Slco1a6	NO
Nat8	NO	Ugt3a1	NO

Gene	In heatmap	Gene	In heatmap
Acox1	YES	Slc6a20b	NO
Cyp2e1	YES	Eci3	YES
Slc22a8	YES	Acox3	NO
Folr1	NO	Mpv17l	NO
Defb29	YES	Hsd3b2	NO
Hao2	NO	Them7	NO
Acaa1b	YES	Lap3	YES
Hykk	NO	Tmem106a	NO
Fth1	NO		

Supplemental Table 5. List of proximal tubule cell specific markers (1) that were included in the analysis to generate the heatmap in Supplemental Figure 1. All genes were not expressed in the samples. For each gene it is indicated if the gene is expressed or not in the samples.

Gene	In heatmap
Gsta2	YES
Agxt2	YES
Cyp2e1	YES
Cryl1	YES
Glyat	NO
Sord	NO
Pdzk1	YES
Upb1	YES
Sod3	YES
Hnf4a	NO

Supplemental Table 6. List of proximal tubule cell specific markers (2) that were included in the analysis to generate the heatmap in Supplemental Figure 2. All genes were not expressed in the samples. For each gene it is indicated if the gene is expressed or not in the samples.

Gene	Segment	In heatmap	Gene	Segment	In heatmap
Ank3	S1	YES	Akr1c21	S2	NO
Ankrd12	S1	YES	Atox1	S2	YES
Apoe	S1	NO	Atp5d	S2	NO
Car12	S1	NO	Atp5e	S2	NO
Ccdc141	S1	YES	Atp5g1	S2	NO
Chrna4	S1	NO	Atp5h	S2	NO
Cpeb3	S1	NO	Atp5j	S2	NO
Cttnbp2	S1	NO	Atp5j2	S2	NO
Cyp24a1	S1	YES	Atp5k	S2	NO
Cyp2d26	S1	NO	Atp5l	S2	NO
Dpep1	S1	NO	Atp5o	S2	NO
Fam13a	S1	NO	Atp6v1f	S2	YES
Gatm	S1	YES	Atp6v1g1	S2	YES
Gm26917	S1	NO	Atpif1	S2	NO
Gm42418	S1	NO	Calml4	S2	YES
Gpx3	S1	YES	Cda	S2	NO
Igfbp5	S1	YES	Cela1	S2	YES
Itch	S1	NO	Chchd10	S2	YES
Itpr2	S1	YES	Chchd2	S2	YES
Kcnq1ot1	S1	NO	Cndp2	S2	NO
Kif12	S1	NO	Cox5a	S2	YES
Lipa	S1	NO	Cox5b	S2	YES
Maf	S1	YES	Cox6b1	S2	NO
Malat1	S1	NO	Cox6c	S2	YES
Neat1	S1	NO	Cox7a2l	S2	YES
Nox4	S1	NO	Cox7b	S2	NO
Ogt	S1	NO	Cyb5a	S2	YES
PISD	S1	NO	Cycs	S2	NO
Prodh2	S1	YES	Cystm1	S2	YES
Ptprd	S1	NO	Dbi	S2	YES
Rsrp1	S1	YES	Ddt	S2	YES
Slc22a29	S1	NO	Edf1	S2	YES
Slc34a1	S1	YES	Eef1a1	S2	YES
Slc34a3	S1	NO	Eif1	S2	YES
Slc3a2	S1	YES	Eif5a	S2	NO
Slc43a2	S1	NO	Etfb	S2	YES
Slc4a4	S1	YES	Fau	S2	NO
Slc5a12	S1	YES	Fth1	S2	NO
Slc5a2	S1	YES	Ftl1	S2	YES
Slc6a19	S1	YES	Fxyd2	S2	NO
Slc7a7	S1	NO	Gabarap	S2	YES
Slc7a8	S1	YES	Gm10076	S2	NO
Snhg11	S1	NO	Gng5	S2	YES
Spp1	S1	NO	Gpx1	S2	YES
Spp2	S1	YES	Gpx4	S2	NO
Steap2	S1	NO	Hint1	S2	YES
Tnfaip8	S1	NO	Hint2	S2	YES
Xist	S1	NO	Hist1h2bc	S2	NO
2010107E04Rik	S2	NO	Hrsp12	S2	NO
2410015M20Rik	S2	NO	Hspe1	S2	YES
Acot13	S2	YES	Iscu	S2	YES
Akr1a1	S2	YES	Mif	S2	NO

Gene	Cell-type	In heatmap	Gene	Cell-type	In heatmap
Mpc1	S2	YES	Rpl39	S2	YES
Mrpl12	S2	YES	Rpl4	S2	YES
Msrb1	S2	YES	Rpl41	S2	YES
Myl6	S2	NO	Rpl5	S2	NO
Naca	S2	NO	Rpl6	S2	NO
Ndufa12	S2	YES	Rpl7	S2	YES
Ndufa2	S2	YES	Rpl8	S2	YES
Ndufa4	S2	YES	Rplp1	S2	YES
Ndufa5	S2	YES	Rplp2	S2	YES
Ndufa6	S2	YES	Rps11	S2	YES
Ndufab1	S2	NO	Rps13	S2	YES
Ndufb2	S2	YES	Rps14	S2	YES
Ndufb4	S2	YES	Rps15a	S2	NO
Ndufc1	S2	YES	Rps16	S2	YES
Ndufs6	S2	YES	Rps17	S2	YES
Ndufv3	S2	NO	Rps2	S2	YES
Nme1	S2	NO	Rps20	S2	YES
Nop10	S2	YES	Rps23	S2	YES
Oaz1	S2	NO	Rps24	S2	YES
Park7	S2	YES	Rps25	S2	YES
Pcbd1	S2	NO	Rps27a	S2	NO
Pfn1	S2	YES	Rps3a1	S2	NO
Ppia	S2	YES	Rps4x	S2	YES
Prdx1	S2	YES	Rps5	S2	NO
Prdx5	S2	YES	Rps6	S2	YES
Romo1	S2	YES	Rps7	S2	YES
Rpl10	S2	YES	Rps8	S2	YES
Rpl11	S2	NO	S100a1	S2	YES
Rpl13a	S2	YES	Sec61g	S2	YES
Rpl14	S2	YES	Tceb2	S2	NO
Rpl17	S2	YES	Timm13	S2	YES
Rpl18a	S2	YES	Timm8b	S2	YES
Rpl19	S2	YES	Tmem256	S2	YES
Rpl21	S2	YES	Tpt1	S2	YES
Rpl22	S2	YES	Ttc36	S2	YES
Rpl23	S2	YES	Txn1	S2	YES
Rpl23a	S2	YES	Txndc17	S2	YES
Rpl24	S2	YES	Ubb	S2	YES
Rpl26	S2	YES	Uqcr10	S2	YES
Rpl27	S2	YES	Uqcr11	S2	NO
Rpl28	S2	YES	Uqcrb	S2	YES
Rpl3	S2	YES	Uqcrfs1	S2	YES
Rpl30	S2	YES	Uqcrh	S2	YES
Rpl31	S2	YES	Uqcrq	S2	NO
Rpl32	S2	YES	Usmg5	S2	NO
Rpl34	S2	NO	Ybx1	S2	NO
Rpl35	S2	YES	Atp11a	S3	NO
Rpl35a	S2	NO	BC005561	S3	NO
Rpl36a	S2	YES	Ces1d	S3	YES
Rpl36al	S2	NO	Ces1f	S3	NO
Rpl37	S2	YES	Cyp2e1	S3	YES
Rpl38	S2	NO	Cyp4a10	S3	NO

Gene	Cell-type	In heatmap	Gene	Cell-type	In heatmap
Cyp4b1	S3	YES	Slc13a3	S3	YES
Fmo5	S3	YES	Slc17a3	S3	YES
G0s2	S3	YES	Slc22a12	S3	NO
Ggt1	S3	YES	Slc22a28	S3	NO
Gm11128	S3	NO	Slc22a30	S3	NO
Kap	S3	NO	Slc22a6	S3	NO
Lpl	S3	YES	Slc27a2	S3	YES
Ly6a	S3	NO	Slc47a1	S3	NO
Mat2a	S3	YES	Slc5a8	S3	YES
Mep1a	S3	YES	Slc6a18	S3	NO
Mlxipl	S3	YES	Slc7a13	S3	YES
Napsa	S3	YES	Slco3a1	S3	YES
Nat8	S3	NO	Tmem252	S3	YES
Nudt19	S3	NO	Tmigd1	S3	YES
Pde4d	S3	YES	Trim7	S3	NO
Polr3e	S3	NO	Ugt3a2	S3	NO
RP23-306P12.3	S3	NO	Zbtb20	S3	YES

Supplemental Table 7. List of proximal tubule segment specific markers (1) that were included in the analysis to generate the heatmap in Supplemental Figure 3. All genes were not expressed in the samples. For each gene it is indicated in which proximal tubule segment the gene is expressed and if the gene is expressed or not in the samples.

Gene	Segment	In heatmap
Nme4	S1	YES
Apoe	S1	NO
Slc5a2	S1	YES
Slc5a1	S2	YES
Slco1a6	S2	NO
Hgd	S2	YES
Slc17a3	S2	YES
Osgin1	S2	NO
Hsd11b1	S2	YES
Serpinf2	S2	YES
Kap	S2	NO
Haao	S2	NO
Slc7a13	S2	YES
Ppic	S2	YES
Tff3	S3	YES
Gng13	S3	NO
Slc23a3	S3	NO
Gc	S3	NO
Cacng5	S3	NO
Tgfbi	S3	YES
Slc22a13	S3	NO
Rbp4	S3	NO

Supplemental Table 8. List of proximal tubule segment specific markers (2) that were included in the analysis to generate the heatmap in Supplemental Figure 4. All genes were not expressed in the samples. For each gene it is indicated in which proximal tubule segment the gene is expressed and if the gene is expressed or not in the samples.

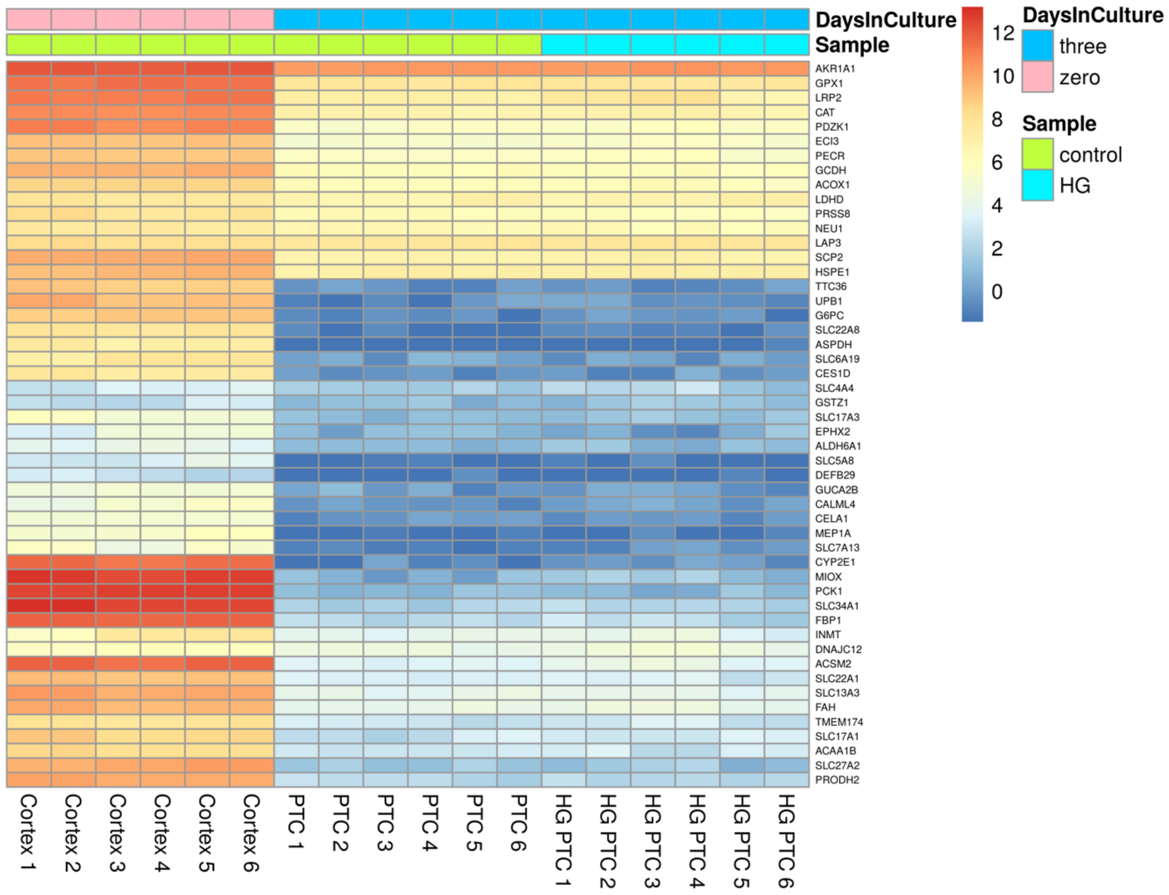

Supplemental Figure 1. Heatmap for proximal tubular cell (PTC) specific markers from Supplemental Table 5. We compare 3 days old cell cultures of primary PTC with cells retrieved directly from the outer renal cortex and between PTC exposed to 15 mM of glucose and control for 8 hours. Genes were clustered using complete-linkage hierarchical clustering. Gene expression goes from blue (low expression) to red (high expression). Samples are ordered by days in culture (DaysInCulture) and by control and high glucose (HG).

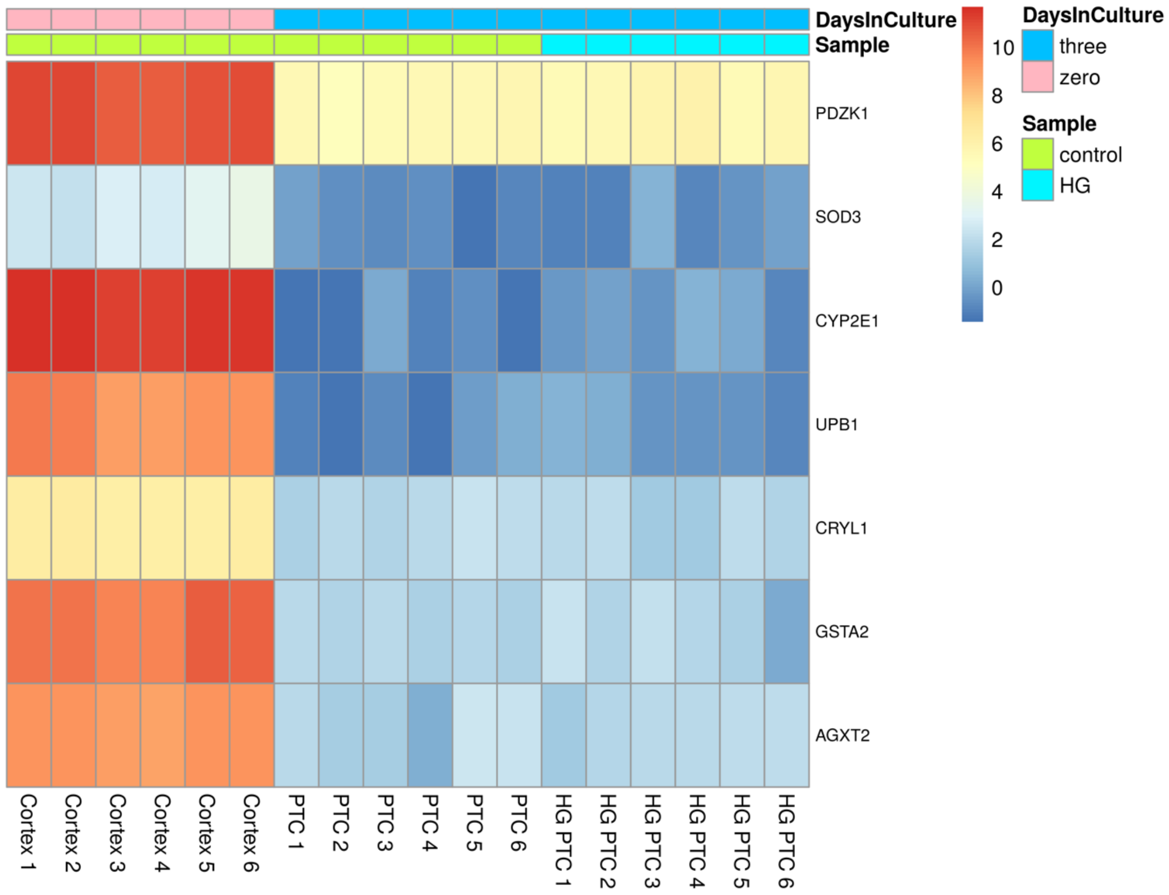

Supplemental Figure 2. Heatmap for proximal tubular cell (PTC) specific markers from Supplemental Table 6. We compare 3 days old cell cultures of primary PTC with cells retrieved directly from the outer renal cortex and between PTC exposed to 15 mM of glucose and control for 8 hours. Genes were clustered using complete-linkage hierarchical clustering. Gene expression goes from blue (low expression) to red (high expression). Samples are ordered by days in culture (DaysInCulture) and by control and high glucose (HG).

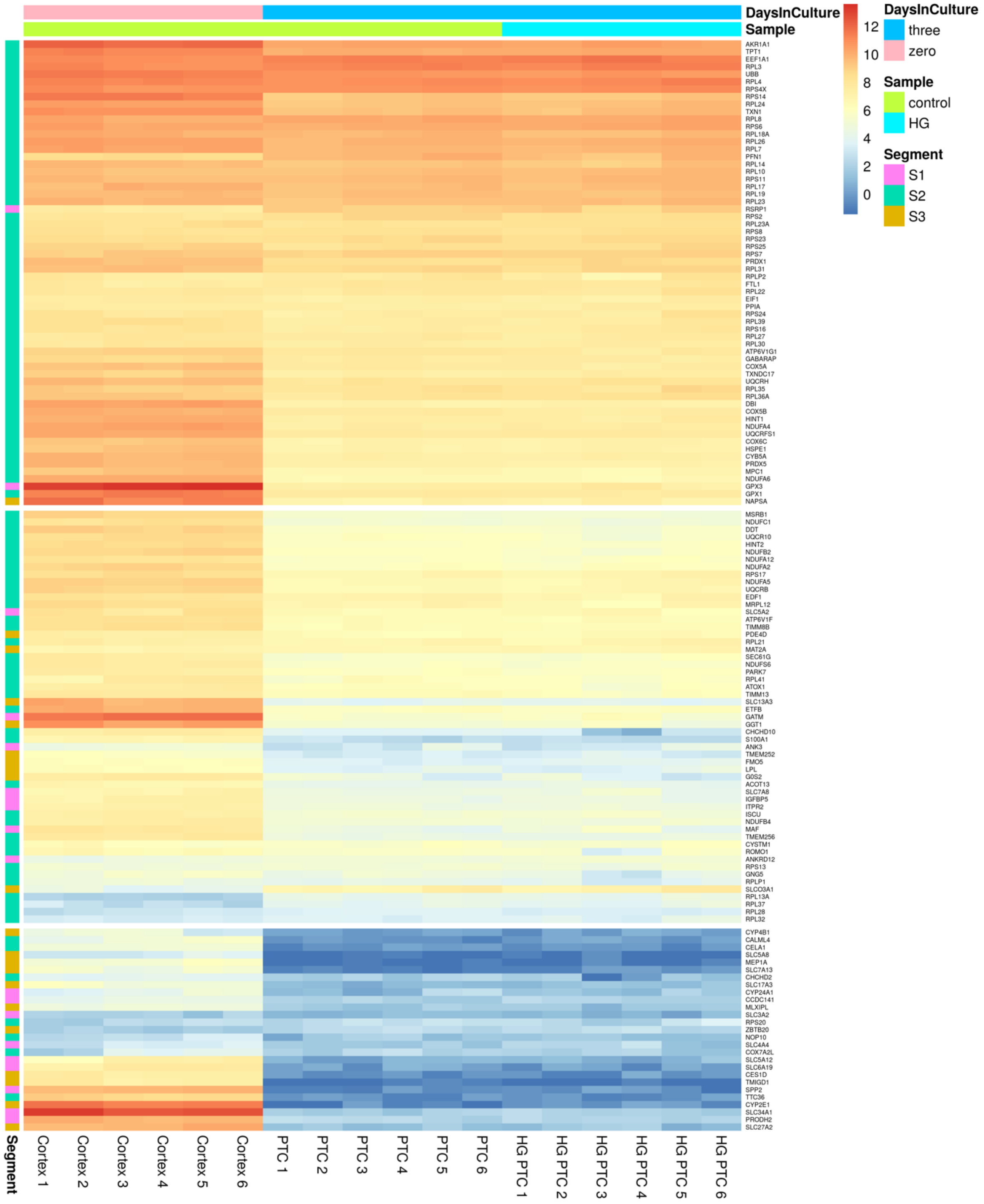

Supplemental Figure 3. Heatmap for proximal tubular cell (PTC) segment specific markers from Supplemental Table 7. We compare 3 days old cell cultures of primary PTC with cells retrieved directly from the outer renal cortex and between PTC exposed to 15 mM of glucose and control for 8 hours. Gene expression goes from blue (low expression) to red (high expression). Genes are clustered using complete-linkage hierarchical clustering and they are classified depending to what proximal tubule segment they belong to. Samples are ordered by days in culture (DaysInCulture) and by control and high glucose (HG).

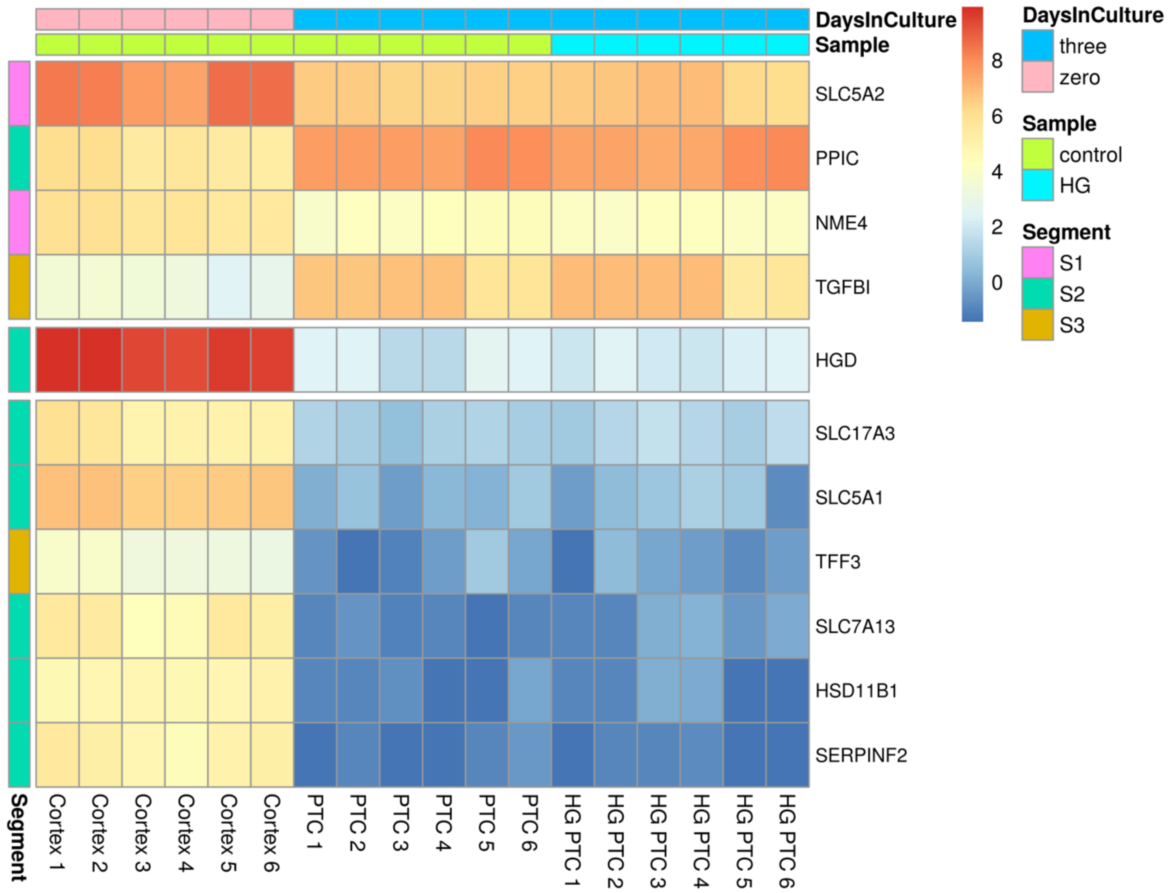

Supplemental Figure 4. Heatmap for proximal tubular cell (PTC) segment specific markers from Supplemental Table 8. We compare 3 days old cell cultures of primary PTC with cells retrieved directly from the outer renal cortex and between PTC exposed to 15 mM of glucose and control for 8 hours. Gene expression goes from blue (low expression) to red (high expression). Genes are clustered using complete-linkage hierarchical clustering and they are classified depending to what proximal tubule segment they belong to. Samples are ordered by days in culture (DaysInCulture) and by control and high glucose (HG).

References

1. Park, J. et al. Single-cell transcriptomics of mouse kidney reveals potential cellular targets of kidney disease. *Science* **360**, 758-763 (2018).
2. Clark, J. Z. et al. Representation and relative abundance of cell-type selective markers in whole-kidney RNA-Seq data. *Kidney Int.* **95**, 787-796 (2019).
